# *Ankrd31* in Sperm and Epididymal Integrity

**DOI:** 10.3389/fcell.2021.741975

**Published:** 2021-11-08

**Authors:** Francesco Manfrevola, Guillaume Martinez, Charles Coutton, Domenico Rocco, Karine Reynaud, Yves Le Vern, Pascal Froment, Linda Beauclair, Denise Aubert, Riccardo Pierantoni, Rosanna Chianese, Florian Guillou

**Affiliations:** ^1^Dipartimento di Medicina Sperimentale, Università degli Studi della Campania L. Vanvitelli, Naples, Italy; ^2^Hôpital Couple-Enfant, Centre Hospitalier Universitaire de Grenoble, UM de Génétique Chromosomique, Grenoble, France; ^3^Genetic Epigenetic and Therapies of Infertility, Institute for Advanced Biosciences INSERM U1209, CNRS UMR 5309, Grenoble, France; ^4^CNRS, IFCE, INRAE, Université de Tours, PRC, Nouzilly, France; ^5^INRAE, Université de Tours, ISP, Nouzilly, France; ^6^Univ Lyon, ENS de Lyon, INRAE, CNRS, Institut de Génomique Fonctionnelle de Lyon, Lyon, France

**Keywords:** ankyrins, spermatogenesis, sperm quality, blood-epididymal-barrier, male infertility

## Abstract

Ankyrin proteins (ANKRD) are key mediators linking membrane and sub-membranous cytoskeletal proteins. Recent findings have highlighted a new role of ANKRD31 during spermatogenesis, elucidating its involvement in meiotic recombination and male germ cell progression. Following testicular differentiation, spermatozoa (SPZ) enter into the epididymis, where they undergo several biochemical and enzymatic changes. The epididymal epithelium is characterized by cell-to-cell junctions that are able to form the blood-epididymal barrier (BEB). This intricate epithelial structure provides the optimal microenvironment needed for epididymal sperm maturation. To date, no notions have been reported regarding a putative role of ANKRD31 in correct BEB formation. In our work, we generated an *Ankrd31* knockout male mouse model (*Ankrd31^–/–^*) and characterized its reproductive phenotype. *Ankrd31^–/–^* mice were infertile and exhibited oligo-astheno-teratozoospermia (a low number of immotile SPZ with abnormal morphological features). In addition, a complete deregulation of BEB was found in *Ankrd31^–/–^*, due to cell-to-cell junction anomalies. In order to suggest that BEB deregulation may depend on *Ankrd31* gene deletion, we showed the physical interaction among ANKRD31 and some epithelial junction proteins in wild-type (WT) epididymides. In conclusion, the current work shows a key role of ANKRD31 in the control of germ cell progression as well as sperm and epididymal integrity.

## Introduction

Beyond the testis, spermatozoa (SPZ) travel into a long-convoluted tube, the epididymis, consisting of three main regions (*caput, corpus*, and *cauda*). Sperm maturation during the epididymal transit consists of impressive morphological, biochemical, and physiological changes at the level of surface protein distribution level, including enzymatic activity, acquisition of motility, migration of the cytoplasmic droplet, and change in sperm transcriptome and proteome (Binato de Souza et al., 2017; Gervasi and Visconti, 2017; Conine et al., 2018; James et al., 2020). In doing so, a continuous interaction with the epididymal luminal microenvironment is required, in the complete absence of *de novo* gene transcription or protein translation. Luminal fluids, collected from the epididymis, show a high degree of compositional diversity, with a substantial segment-to-segment variation in inorganic ions, proteins, and small non-coding ribonucleic acid (RNA) transcripts that contribute to the regionalized functionality of this long tubule (Zhou et al., 2018). As a consequence, sperm dysfunctions should not be interpreted as just the result of defective spermatogenesis, but also as an inadequate epididymal maturation process due to improper epididymal function (Elbashir et al., 2021).

The epididymal epithelium is composed of several epithelial cell types, such as clear cells, principal cells, basal cells, and narrow cells, with specific morphological and functional features (Breton et al., 2019). Epithelial cells establish an intricate communication network mediated by cell-to-cell junctions that are able to form the blood-epididymal barrier (BEB), a well-defined structure that protects the luminal microenvironment from pathogen invasion (Cornwall, 2009; Breton et al., 2016). The BEB also promotes epididymal lumen acidification, indispensable for: (i) sperm maturation, (ii) epididymosome transfer from epithelial cells to SPZ, and (iii) maintenance of sperm quiescence during epididymal transit (Sullivan, 2015; Zhou et al., 2018).

Epithelial intercellular junction complexes forming BEB include tight junctions, adherens junctions, and gap junctions.

Tight junctions between adjacent principal cells are the most prevalent intercellular junctions present in the epididymal epithelium and are involved in the impermeable features of BEB. Several transmembrane proteins interact with each other to form tight junctions, including occludins (OCLN), claudins (CLDN), and intracellular zona occludens proteins (ZO) (Dubé et al., 2010).

Adherens junctions, formed by cadherins and nectins, participate in cell adhesion and stabilization of BEB, acting as intracellular signaling modulators. The prevalent isoform of cadherin produced in rodent epididymis is E-cadherin (CDH1) (Cyr et al., 1992). As tight junction proteins, the cytoplasmatic domain of cadherins interacts with other proteins, including catenin, actin, and ZO-1 to form protein complexes able to link cadherins to the cytoskeleton (Cyr et al., 2007).

Gap junctions, formed by connexin proteins, appear localized between principal cells and basal cells or basal cells and clear cells, and are fundamental for intercellular communication. Connexin43 (Cx43) was the first connexin identified in the epididymis (Cyr, 2011).

Ankyrins (ANKRD) are a family of proteins that, through specific structural domains, act as a link between membrane proteins and sub-membranous cytoskeletal proteins such as actin and β-spectrin. Ankyrin-cytoskeleton protein complexes are involved in several cellular functions including: (i) cellular adhesion, and (ii) cellular cytoarchitecture modulation (Cunhaa and Mohlera, 2009).

Recently, new findings regarding the involvement of ANKRD in male reproduction have been reported through the knockout mouse model of *Ankrd31* (*Ankrd31^–/–^*), where *Ankrd31* gene deletion causes meiotic arrest and sterility (Boekhout et al., 2019; Acquaviva et al., 2020). Specifically, in spermatocytes, ANKRD31 participates to homologous recombination acting as a scaffold protein that anchors several factors needed for correct double-strand break formation.

In addition, in recent years, several studies have suggested a potential role for ANKRD in stabilizing intercellular junctions. ANKRD-G interacts with CDH1, and its binding with β-2-spectrin induces CDH1 accumulation at the lateral membrane of epithelial cells (Kizhatil et al., 2007). In addition, ANKRD-G interacts with the cytoplasmic tail of vascular endothelial cadherin (VE-cadherin) stabilizing it at the cell surface (Cadwell et al., 2016). The involvement of ANKRD in broadly facilitating the organization of various junction types—working as a scaffolding platform, a central node inside an interactome sustaining junction integrity—has also been evaluated in tight or gap junctions (Rajasekaran et al., 2008; Naydenov and Ivanov, 2011). In this regard, ANKRD-G is able to modulate Cx43 expression at myocyte-level junctions (Sato et al., 2011; Delmar, 2012).

Based on these interconnections, in the current work, we reported the generation of an *Ankrd31* knockout male mouse model (*Ankrd31^–/–^)* by using a clusters of regularly interspaced short palindromic repeats (CRISPR)/Cas9 genome editing strategy against exon 4 of the *Ankrd31* gene. *Ankrd31^–/–^* reproductive phenotype was, therefore, characterized, at the testicular, sperm, and epididymal level. In detail, *Ankrd31* gene deletion induced a significant reduction in testis weight, a disorganized seminiferous epithelium, and an anomalous germ cell progression with a deficit in post-meiotic cell differentiation. In fact, *Ankrd31^–/–^* showed a significant reduction in spermatid number and increase in spermatogonia and spermatocyte number. Interestingly, SPZ were few and displayed morphological abnormalities and lack of motility, that is a male infertility phenotype of oligo-astheno-teratozoospermia.

In addition, we analyzed, at the molecular level, the integrity of BEB in *Ankrd31^–/–^*, focusing our attention on the expression of intercellular junction modulators. Interestingly, a complete deregulation of epididymal tight, adherens, and gap junctions was found. Subsequently, with the aim of understanding whether the altered intercellular junction profile of BEB depended on *Ankrd31* gene deletion, protein immunoprecipitation experiments in wild-type (WT) epididymides were carried out to investigate a possible physical interaction between ANKRD31 and some epithelial junction proteins.

In conclusion, we demonstrated that *Ankrd31* gene deletion impaired spermatogenesis, producing sperm cell damage and altering epididymal maturation by affecting the integrity of BEB. Thus, we hypothesized that protein interaction deregulation in BEB not only negatively affects the normal development of epithelial intercellular junction complexes, but may also contribute to sperm dysfunction.

## Materials and Methods

### *Ankrd31^–/–^* Male Mouse Model

*Ankrd31* mutant lines were generated using CRISPR/Cas9 genome editing (Hwang et al., 2013), targeting exon 4 of the *Ankrd31* gene. In order to produce a small frameshift deletion, two guide RNAs were designed using the online platform at http://crispor.tefor.net/. For each CRISPR-Cas9 ribonuclear protein (RNP) complex, fifty nanograms per microliter of Cas9 protein (PNA Bio) was complexed with an equimolar amount of crRNA (Eurogentec - ANKRDd315 5′[C^∗^A^∗^U^∗^]UCUCCCGAGAUUCAACUGUUUUAGAGCUAU GCUGUUUU3′ or ANKRDd313 5′ [G^∗^U^∗^U]GUUGCUGGCUC UUAGUGGUUUUAGAGCUAUGCUGUUUUG3′) and tracrRNA (TriLink) in microinjection buffer (Life Technologies), as previously described (Teixeira et al., 2018). The two RNP complexes were co-injected into the cytoplasm of C57BL/6JCrl fertilized oocytes. The oocytes were subsequently transferred into pseudopregnant recipients. Design, production of RNP complexes, injections, and embryo transfer were performed by the AniRA-PBES transgenic facility of SFR Biosciences, Lyon. Out of 189 injected embryos, 90 were transferred into females and 14 were born. Eleven of the 14 born pups had alterations in the targeted genomic locus. Two mice that were heterozygotes for predicted frameshift-causing alleles (mut1 and mut2) were bred with C57BL/6JCrl wild-type (WT) mice to establish mouse lines. All experiments reported in the manuscript are based on samples from mice that were derivatives of the founder lines after at least three backcrosses.

Animal care and experimental procedures were performed following French and European guidelines on the protection of animals used for scientific purposes and approved by an ethical committee for animal experimentation (CEEA Val de Loire Projet 12884).

### Total RNA Preparation

Trizol R Reagent (Invitrogen Life Technologies, Paisley, United Kingdom) was used to extract total RNA from WT and *Ankrd31^–/–^* mouse epididymides following the manufacturer’s instructions. In brief, the epididymis was homogenized in Trizol Reagent and incubated for 5 min at 20°C. Then, 0.2 ml chloroform/ml Trizol Reagent was added and the samples were centrifuged at 12,000× g for 15 min at 4°C. The aqueous phase was transferred to a fresh tube and total RNA was precipitated by mixing with isopropyl alcohol (0.5 ml/ml Trizol Reagent) and 1 μl of glycogen (20 mg/ml). After centrifugation at 12,000× g for 10 min at 4°C, the RNA pellet was washed with 75% ethanol, centrifuged at 7,500× g for 10 min at 4°C, and dissolved in DEPC-H_2_O. Total RNAs were assessed with a NanoDrop 2000 spectrophotometer (Thermo Fisher Scientific, Waltham, MA, United States) to quantify concentration (ng/ml) and purity (260/280 and 260/230 ratios). Then, RNA aliquots (10 μg) were treated with 2U DNase I (RNase-free DNase I, Ambion, Thermo Fisher Scientific, Massachusetts, United States) to remove potential contamination of genomic DNA and finally preserved at −80°C until the next step.

### RNA Expression Analysis by One-Step Evagreen qRT-PCR

According to the manufacturer’s instructions, a kit containing quantitative real-time polymerase chain reaction (qRT-PCR) enzyme mix and an Evagreen qPCR Mastermix (Applied Biological Materials Inc.) was used for gene expression analysis in WT and *Ankrd31^–/–^* epididymides (*n* = 5 animals for each genotype). A concentration of 50 ng of total RNA was used for all reactions on a CFX-96 Real Time polymerase chain reaction (PCR) System (Biorad). A negative control, without RNA, was included. The qRT-PCR in triplicates from each genotype was analyzed. Gene expression analysis, corrected for PCR efficiency, and normalized toward the reference gene (*RP18S*), was performed by CFX Manager software (Bio-Rad). Normalized fold expression (n.f.e) of mRNAs was calculated by applying the 2^–ΔΔCt^ method.

### PCR Primer Design

Primers to amplify selected RNAs in WT and *Ankrd31^–/–^* samples were designed through the online tool Primer-BLAST^1^. Primers for mouse genes are shown in Table 1.

**TABLE 1:** Primer sequences and annealing temperatures.

Gene primers	Sequences 5′–3′	Tm (°C)
*Ocln* S	cctccacccccatctgacta	58
*Ocln* AS	cagcagcagccatgtactct	
*ZO-1* S	gcttctcttgctggccctaa	58
*ZO-1* AS	tactctgagatgggggtggg	
*Cldn5* S	tgccggtgtcacagaagtac	56
*Cldn5* AS	ttctccagctgccctttcag	
*CDH1* S	atcagctgccccgaaaatga	54
*CDH1* AS	cttgaaggtcagcagcctga	
*Cx43* S	gtacccaacagcagcagact	54
*Cx43* AS	ctgcttcaggtgcatctcca	
*RP18S* S	gagactctggatgctaactag	56
*RP18S* AS	ggacatctaagggcatcacag	
*Ankrd31* S	catatatgctaatggtaccctacca	53
*Ankrd31* AS	ccttgtaattagtaatttgccacag	

### Protein Extraction and Western Blot Analysis

The epididymis from WT and *Ankrd31^–/–^* mice (*n* = 5 animals for each genotype) was separately homogenized in RIPA buffer [PBS, pH 7.4, 10 mM of dithiothreitol, 0.02% sodium azide, 0.1% SDS, 1% NP-40, 0.5% sodium deoxycholate, in the presence of protease inhibitors (10 μg/ml of leupeptin, aprotinin, pepstatin A, chymostatin, and 5 μg/ml of TPCK)] and sonicated three times for 30 s bursts, each at 60 mW. Proteins were separated by SDS-PAGE (8% acrylamide) and transferred to a polyvinylidene difluoride membrane (GE Healthcare) at 280 mA for 2.5 h, at 4°C. The filters were treated for 2.5 h with blocking solution [5% non-fat milk, 0.25% Tween-20 in Tris-buffered saline (TBS, pH 7.6)] and incubated with different primary antibodies [occludin, diluted 1:500, sc-133255 Santa Cruz Biotechnology, Cambridge, United Kingdom; tubulin, diluted 1:5000, Sigma-Aldrich (T5168); actin, diluted 1:2000, sc-517582 Santa Cruz Biotechnology, Cambridge, United Kingdom; ZO-1, diluted 1:500, sc-33725 Santa Cruz Biotechnology, Cambridge, United Kingdom; claudin5 diluted 1:500, 4C3C2, Invitrogen; E-cadherin, diluted 1:500, SHE78-7, Invitrogen; connexin43; diluted 1:500; sc-271837 Santa Cruz Biotechnology, Cambridge, United Kingdom; SPECTRIN, diluted 1:500; sc-53444 Santa Cruz Biotechnology, Cambridge, United Kingdom] in TBS-milk buffer (TBS pH 7.6, 3% non-fat milk) overnight, at 4°C. The filters were washed in 0.25% Tween20-TBS and incubated with 1:1,000 horseradish peroxidase-conjugated mouse IgG (Dako Corp., Milan, Italy) in TBS-milk buffer and then washed again. An enhanced chemiluminescence-Western blotting detection system (Amersham ECL Western Blotting Detection Reagent, cod: RPN2106, GE Healthcare) was used to detect the immune complexes. Western blot experimental triplicates from each genotype analyzed (*n* = 5 animals for each genotype) were quantified by densitometry analysis, adjusted relatively to Ponceau S staining, and reported as OD fold change (mean ± SEM).

### ANKRD31 Antibody

ANKRD31 antibodies were produced in rabbits by injection of two specific peptide regions of ANKRD31 protein. The protein sequences of ANKRD31 of different species (cattle, human, horses, rat) were compared to identify the specific regions for mouse ANKRD31. We, then, identified two potentially immunogenic peptides that were used to generate the antibodies. The sequences of peptides are: SLERKQDTDKNYTKKGP and NPKRRNKKTASQQPSAG.

The peptides were coupled to keyhole limpet hemocyanin (KLH) and injected into rabbits. Four immunization series were realized (0, 7, 14, and 34 days). The antibodies against ANKRD31 were detected in serum at 42 days after the last immunization. The specificity of the ANKRD31 antibody was verified by Western blot, immunochemistry, and protein immunoprecipitation (IP).

### Protein Immunoprecipitation Assay

For IP, the WT epididymis was lyzed in RIPA buffer, in the presence of protease inhibitors (10 μg/ml of leupeptin, aprotinin, pepstatin A, chymostatin, and 5 μg/ml of TPCK), sonicated three times for 30 s bursts, each at 60 mW, and then incubated on ice for 30 min. After centrifugation at maximum speed for 30 min at 4°C, the protein supernatant was collected. A concentration of 500 mg of supernatant proteins from the WT epididymis was incubated with 2 μg of ANKRD31 antibody or IgG as negative control (Sigma-Aldrich-12370) under rotary agitation overnight at 4°C. Following, Protein A/G PLUS Agarose Beads (Santa Cruz Biotechnology- sc-2003) were added to each sample and incubated overnight under rotary agitation at 4°C. After bead incubation, samples were washed three times (3,000× g for 3 min at 4°C) in TBS pH 7.6 and boiled in Laemmli sample buffer for 10 min to be later analyzed by SDS-PAGE.

### Histology and Immunocytochemistry Analysis

WT and *Ankrd31^–/–^* mouse testes (*n* = 5 for each genotype) were fixed overnight in Bouin’s solution, dehydrated in ethanol, cleared in xylene, and embedded in paraffin using standard procedures. Microtome serial sections (7 μm thick) were cut and processed for histopathological analysis. For hematoxylin and eosin staining (H&E), sections were deparaffinized and processed using standard procedures. Histological observations and analysis were conducted under a light microscope (Leica CTR500, Leica Microsystems Inc., Milan, Italy) and images were captured using a high resolution digital camera (Leica DC300F). For DAPI immunofluorescence staining, testis sections were deparaffinized, rehydrated, and permeabilized with PBS pH 7.4 containing 0.1% Triton-X-100. Cell nuclei were marked with DAPI and the analysis was conducted under an optical microscope (Leica DM 5000 B + CTR 5000) with a UV lamp. For immunocytochemistry staining, WT and *Ankrd31^–/–^ caput* and *cauda* epididymal (*n* = 5 for each genotype) sections were deparaffinized, rehydrated, and permeabilized with PBS pH 7.4 containing 0.1% Triton-X-100. A citrate buffer of 0.01 M (pH 6.0) was used for antigen retrieval. After blocking with PBS containing 5% BSA and normal goat serum (diluted 1:5), sections were incubated with anti-ANKRD31 antibody (diluted 1:100) overnight at 4°C. Immunoreactivity was revealed using the avidin/biotin complex system and H_2_O_2_/DAB as the substrate/chromogen. The histological observations and analysis were conducted under a light microscope (Leica CTR500, Leica Microsystems Inc., Milan, Italy) and images were captured using a high resolution digital camera (Leica DC300F). WT and *Ankrd31^–/–^ cauda* sperm Harris-Shorr staining (*n* = 6 for each genotype) was performed using standard procedures.

### Germ Cell Isolation From Testis

The seminiferous tubular cells were isolated from 8-week WT or *Ankrd31^–/–^* mouse testes (*n* = 5 animals for each genotype) according to the following protocol. The tunica albuginea was removed from testes. The testis was incubated for 10 min at 37°C in 3 ml of L15 medium containing 1 ml of collagenase (2 mg/ml) and 20 μl of DNAse 1 (2 mg/ml). The seminiferous tubules were isolated by sedimentation and the supernatant containing cells from the interstitial compartment was removed. The seminiferous tubules were incubated for 15 min at 37°C in 2.45 ml of L15 containing 2 ml of collagenase (2 mg/ml) and 20 μl of DNAse 1 (2 mg/ml). The cells of the seminiferous tubules were recovered by centrifugation at 80× g for 15 min. The cell pellet was suspended and fixed for 15 min in 100 μl of PBS containing 4% of paraformaldehyde. The cells were then centrifuged at 80× g for 8 min. The cell pellet was washed in PBS and centrifuged at 80× g for 8 min.

### Fluorescence-Activated Cell Sorting

Cell analysis and sorting was carried out on WT and *Ankrd31^–/–^* testicular germ cell populations (*n* = 6 animals for each genotype), using a MoFlo Astrios^EQ^ high speed cell sorter (Beckman Coulter Inc., Brea, CA, United States) with a nozzle of 90 μm using Summit 6.3 software (Beckman Coulter Inc.). Analysis was triggered with a blue laser (488 nm), debris was eliminated based on morphological criteria using forward scatter (FSC) vs. side scatter (SSC). We used Hoechst 33342 (Sigma) fluorophore to measure DNA content, excited using a violet laser (405 nm), and the blue fluorescence emitted was measured trough a 448/59 nm band pass filter. To preserve cell quantity for post-sorting experiences, a maximum of 200,000 events was analyzed before proceeding to the gating selection. Three peaks with increasing Hoechst fluorescence, representative of haploid (1C—Region 1), diploid (2C—Region 2), and tetraploid (4C—Region 3) cells, were detected and sorted using a dot-plot of side scatter (SSC) vs. Hoechst blue fluorescence. Sorting flow rate was adapted to the sample and set around 10,000 events/s for the most concentrated samples; for cell recovery, a 1.5 ml Eppendorf tube containing 350 μl of RPMI with 10% FBS was used.

### Sperm Parameters

After sacrifice *via* cervical dislocation, sperm cells from *cauda* epididymides (*n* = 5 animals for each genotype) were allowed to swim for 10 min at 37°C in 1 ml of M2 medium (Sigma-Aldrich, L’Isle d’Abeau, France). Sperm concentration was evaluated with a counting chamber, sperm vitality with eosin-nigrosin staining, and motility analysis was performed using a computational sperm analyzer (Hamilton Thorn Research, Beverley, MA, United States) as previously described (Martinez et al., 2017), with an evaluation of total and progressive motility, curvilinear velocity (VCL, μM/s), straight-line velocity (VSL, μM/s), average path velocity (VAP, μM/s) and lateral amplitude of the head (ALH, μM). A minimum of 100 motile SPZ were recorded in real time and analyzed for each sample. The remaining sperm cells were washed by centrifugation for 5 min at 500× g in PBS and fixed either in ether/ethanol 1:1 for morphology analysis or in 3:1 methanol/acetic acid solution for fluorescence *in situ* hybridization (FISH) analysis. Morphology analysis was performed on at least 200 cells for each sample after the Harris-Shorr staining procedure.

### Chromomycin A3 Staining

After methanol/acetic acid fixation, *cauda* epididymal SPZ (*n* = 6 animals for each genotype) were spread on Superfrost slides and air-dried at room temperature overnight (*n* = 3 for each animal). Cells were stained through incubation in 0.25 mg/ml of chromomycin A3 (CMA3) solution in McIlvaine buffer (pH 7) for 20 min followed by two washes for 2 min with McIlvaine buffer. Slides were then mounted with DAPI II (Abbott Laboratories) to counterstain sperm nuclei. A minimum of 100 sperm cells were evaluated and counted for each assay.

### Aniline Blue Staining

*Cauda* epididymal SPZ (*n* = 6 animals for each genotype) samples were dried on slides (*n* = 3 for each animal). After glutaraldehyde fixation, slides were incubated for 5 min in water, 10 min in 5% aniline blue solution diluted in 4% acetic acid solution, twice for 2 min in water, 2 min in 70, 90, and 100% ethanol solutions and, finally, 2 min in toluene. Slides were then mounted with Eukitt^®^ and analyzed using a microscope with a transmitted light microscope 100× objective with oil. A total of 100 SPZ for each slide were evaluated, and microscopic analysis was performed under an optical microscope (Leica DM 5000 B + CTR 5000). A minimum of 100 sperm cells were evaluated and counted for each assay.

### Sperm FISH and Scoring

Post fixation, sperm cells (*n* = 6 animals for each genotype) were spread onto slides (*n* = 3 for each animal) at optimum density to avoid nuclei overlapping and then dried overnight. Sequentially sperm cells were washed in 2× Standard Saline Citrate (SSC) solution, decondensed with 10 mM of DTT in 0.1 M of Tris-HCl, pH 8.0 for 30 min at room temperature and then washed in SSC solution, and dehydrated with ethanol. Three-color FISH was performed using whole chromosome painting probes for autosome 8 (XMP8 Orange, D1408-050-OR and XMP 8 Green, D-1408-050-FI, MetaSystems Probes), and both sexual chromosomes (XMPX Orange, D-1420-050-OR and XMPY Green, D-1421-050-FI, MetaSystems Probes). Denaturation occurred at 72°C for 5 min and hybridization at 37°C overnight. Slides were then washed for 2 min in 0.4× of SSC solution with 0.1% (v/v) Nonidet-P40 (Sigma-Aldrich) and 1 min in 2× of SSC solution at room temperature. Nuclei were counterstained with DAPI II counterstain medium (Abbott Molecular Inc.). Scoring was performed with a METAFER Metasystems^®^ device, previously validated for sperm FISH analysis (Martinez et al., 2013). The galleries of images provided by the machine were manually verified by two experienced biologists (GM and CC) applying the same strict criteria. A minimum of 5,000 nuclei was analyzed for each assay. Sperm cells were considered normal if they showed one distinct signal for each chromosome, disomic if they showed two distinct signals for a chromosome, and diploid if they showed two distinct signals for all chromosomes investigated.

### Fertility Test

Mating experiments were conducted to evaluate if *Ankrd31* gene deletion negatively impaired male fertility. Copulatory plug formation was monitored as parameter of appropriate mounting behavior. For the analysis, equal numbers of 8-week-old male/female WT mice and male/female *Ankrd31^–/–^* mice were used (*n* = 10 for each sex/genotype). The strategy included three types of mating: (♂WT ×♀WT); (♂WT ×♀*Ankrd31^–/–^*); (♂*Ankrd31^–/–^* ×♀WT). Mouse fertility index was defined evaluating the frequency of pups born for each individual pair of mice and for experimental groups.

### Statistical Analysis

ANOVA followed by Student’s *t*-test (for two independent group comparisons) was conducted to identify groups having different mean. Differences with *p* < 0.05 were considered statistically significant. Data are expressed as the mean ± SEM from at least five independent animals for each genotype or experimental group. For qRT-PCR and Western blot analyses, triplicates from each animal/genotype or experimental group were considered.

## Results

### *Ankrd31^–/–^* Generation Using CRISPR/Cas9 Genome Editing

**FIGURE 1:**
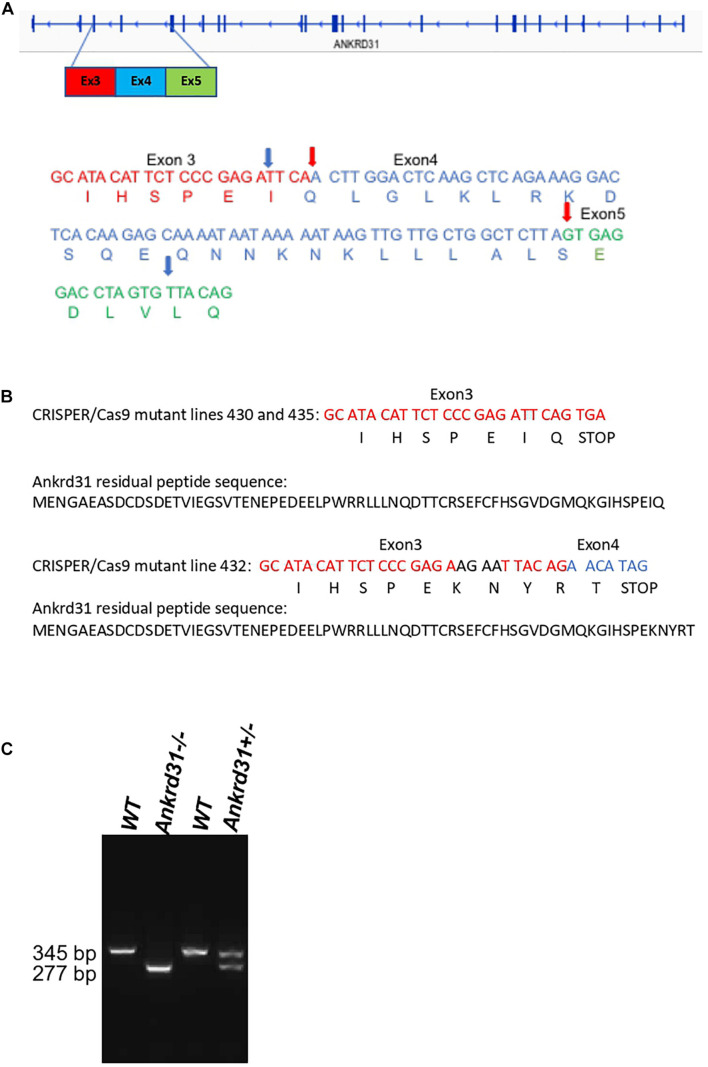
Deletion of exon 4 of the *Ankrd31* gene using CRISPR/Cas9 genome editing. The sequences of the two RNA guides are included in section “Materials and Methods.” **(A)** The entirety of DNA and amino acid sequences of exon 4 (blue) and partial DNA and amino acid sequences of exon 3 (red) and exon 5 (green) are shown in wild type (WT) and in the mutant lines (430, 435, 432). The real cutoff sites observed by sequencing are indicated by red arrows for mutant lines 430 and 435 and blue arrows for mutant line 432. In all mutant lines, a stop codon was generated. **(B)** The residual peptide sequences of the ANKRD31 protein expressed in the mutant lines are shown. **(C)** An example of PCR showing the deletion of exon 4. In WT mice, the amplified fragment was 345 bp. In mice with the deletion of exon 4, the amplified fragment was 277 bp.

*Ankrd31^–/–^* mutant lines were generated using CRISPR/Cas9 genome editing. Our strategy used two guide RNAs for the deletion of exon 4 and generated a frameshift to create a stop codon (Figure 1A). Ninety injected embryos with the two guide RNAs were transferred into females and 14 mice were born. Eleven of the 14 born pups had alterations in the targeted genomic locus. Eight mice showed a single CRISPR/Cas9 cut-off site. Only three mice showed the double cleavage leading to the deletion of exon 4. Sequencing of exons 3 and 4 of the *Ankrd31* gene in three mice are shown in Figure 1B. For lines 430 and 435, the sequences showed that the CRISPR/Cas9 cut exactly occurred as expected from the experimental design. We observed the deletion of exon 4 and the formation of the expected stop codon. In these two lines of mice, a residual peptide of 66 amino acids out of the 1857 amino acids that the ANKRD31 protein possesses should be expressed. The sequence of this peptide is shown in Figure 1B. For line 432, the two guide RNAs slightly slipped, the cuts made by CRIPSR/Cas9 shifted in 5′ by 4 nucleotides upstream of the theoretical cut-off site and in 3′ by 14 nucleotides downstream of the theoretical cut-off site (Figure 1B). This caused a change in reading frame and the stop codon was shifted by 15 nucleotides. In this line of mice, a residual peptide of 66 amino acids added with 5 amino acids without homology with ANKRD31 protein should be expressed. The sequence of this peptide is shown in Figure 1B. An example of the exon 4 deletion from mouse line 430 is shown by PCR (Figure 1C). All experiments reported in the manuscript were based on samples from mice that were derivatives of founder lines after at least three backcrosses. All three lines showed the same phenotypes described in the results below.

### *Ankrd31* Gene Deletion Affects Testicular Morphology

**FIGURE 2:**
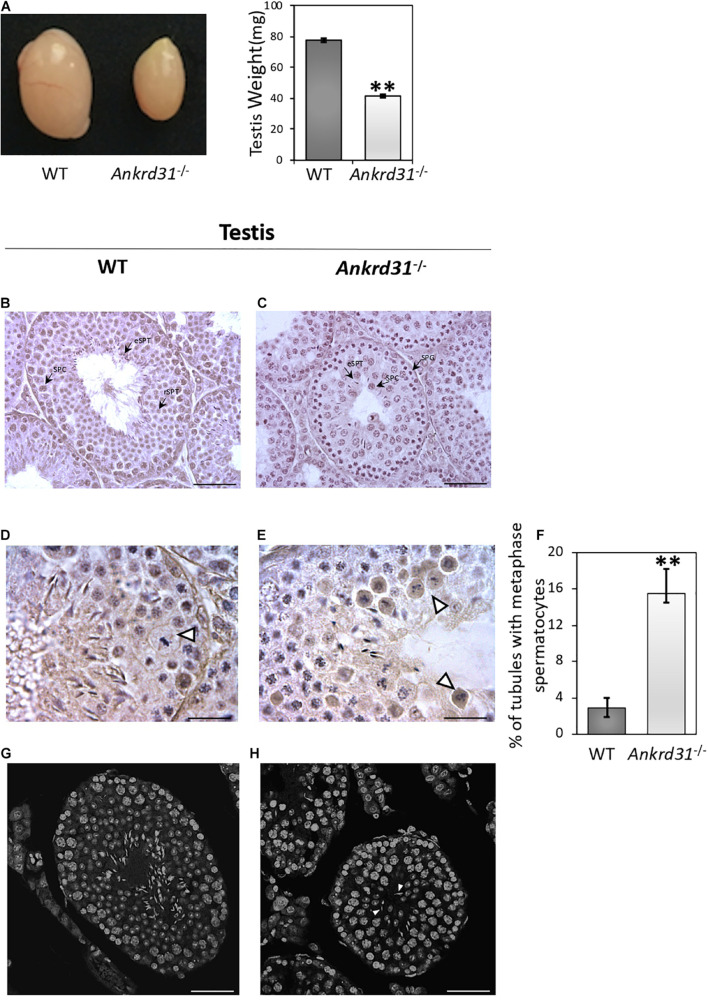
Morphological characterization of *Ankrd31^–/–^* testes. **(A)** Comparative size analysis between WT and *Ankrd31^–/–^* testes. Testis weight (mg) analysis was carried out on WT and *Ankrd31^–/–^* testes (*n* = 20). Data are reported as mean value ± S.E.M. **(B,C)** Defective spermatogenesis was observed by H&E staining of WT and *Ankrd31^–/–^* Bouin’s fixed seminiferous tubule sections (7 μm) collected from 8-week-old testes. SPG, spermatogonia; SPC, spermatocytes; rSPT, round spermatids; eSPT, elongated spermatids (black-arrowheads). **(D,E)** Cells in metaphase are indicated by white arrowheads. **(F)** Quantification of seminiferous tubules containing spermatocytes in metaphase in WT and *Ankrd31^–/–^* testes (*n* = 10 for each genotype). The number of total tubules counted in five different animals for each genotype corresponded to *n* = 650. Data are expressed as percentage (%) of tubules containing cells in metaphase and reported as mean ± SEM, **p < 0.01. **(G,H)** Classification of germ cells in respect to chromatin structure marked with DAPI staining in WT and *Ankrd31^–/–^* testicular sections (7 μm). The elongated spermatids not correctly oriented toward seminiferous lumen are indicated by white arrowheads. **(B–H)** Scale bar corresponds to 50 μm; **(D,E)** scale bar corresponds to 25 μm.

With the aim to investigate the impact of *Ankrd31* gene deletion on spermatogenesis, WT and *Ankrd31^–/–^* male mice were sacrificed, testes were collected and used for histological and molecular analyses. *Ankrd31^–/–^* testes appeared macroscopically smaller than WT. A significant reduction in testis weight (*p* < 0.01), from a media value of 77.5–41.5 mg was, in fact, observed in *Ankrd31^–/–^* compared to WT, suggesting that *Ankrd31* gene deletion negatively affected normal testis development (Figure 2A).

Histological analysis performed by H&E staining on testicular sections showed inefficient spermatogenesis in *Ankrd31^–/–^* testes (Figure 2C). In comparison to spermatogenic stage VIII of WT testes (Figure 2B), *Ankrd31^–/–^* testes showed a complete deregulation of normal seminiferous epithelium morphology. The physiologic basal and abluminal germ cell regionalization was not maintained in *Ankrd31^–/–^* testes, indeed a large number of spermatocytes was found in the proximity of tubular lumen. In addition, WT testis sections showed a complete set of germ cells, including spermatocytes, round spermatids, and elongated spermatids (Figures 2B–D). Conversely, *Ankrd31^–/–^* testis sections showed that a large number of spermatogonia and spermatocytes were not completely differentiated into spermatids, in association with sporadic spermatids not correctly oriented toward the seminiferous lumen (Figure 2H). Cells in metaphase with unaligned chromosomes were frequently observed in *Ankrd31^–/–^* compared to WT testes (Figures 2D,E). In addition, a significant increase of the percentage of seminiferous tubules containing spermatocytes in metaphase was observed in *Ankrd31^–/–^* testes (Figure 2F). Finally, immunofluorescent DNA staining by DAPI revealed an abundance of cells with high DNA content, suggesting an accumulation of early stage meiotic cells (Figures 2G,H).

### *Ankrd31* Gene Deletion Affects Germ Cell Content

**FIGURE 3:**
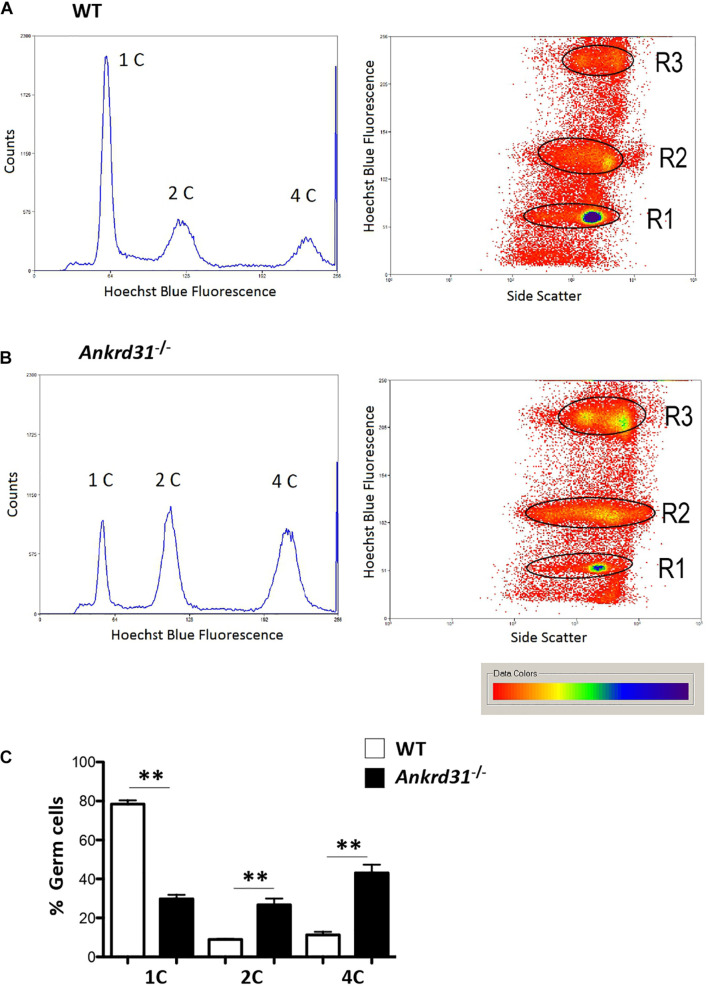
Representative histograms and density plot showing the distribution of testicular germ cell populations, based on their DNA content, in WT and *Ankrd31^–/–^* by flow cytometry. **(A)** FACS analysis of isolated WT male germ cells. **(B)** FACS analysis of isolated *Ankrd31^–/–^* male germ cells. **(C)** Quantification analysis between WT and *Ankrd31^–/–^* germ cell subpopulations isolated from *n* = 6 animals/each genotype. Data are expressed as percentage (%) of germ cells and reported as mean ± SEM, **p < 0.01.

To confirm the morphological studies suggesting impaired spermatogenesis in *Ankrd31^–/–^* testes, we carried out a Fluorescence-Activated Cell Sorting (FACS) analysis to quantify the different germ cell sub-populations in WT and *Ankrd31^–/–^* testes and cells. The inputted single-cell suspensions were obtained by testis dissociation. After that, the single-cell suspension was stained with Hoechst, a fluorescent dye staining DNA, and emitted blue fluorescence after excitation by violet laser at 405 nm. Germ cell analysis data clearly showed the three typical germ cell sub-populations related to DNA fluorescence intensities: 1C, 2C, and 4C, corresponding to spermatids, spermatogonia, and spermatocytes, respectively (Figures 3A,B). The single-plot and dot-plot histograms showed a reduction in 1C peak (spermatid cell population) and an increase in 2C and 4C peaks (spermatogonia and spermatocyte cell populations, respectively) in *Ankrd31^–/–^* testes compared to WT (Figure 3B). Figure 3C showed the percentage of germ cells in WT and *Ankrd31^–/–^* testes: a significant reduction in spermatid content (1C) and a significant increase in spermatogonia and spermatocyte content (2C and 4C, respectively) were observed in *Ankrd31^–/–^* testes compared to WT, suggesting that *Ankrd31* gene deletion negatively affected germ cell progression.

### *Ankrd31* Gene Deletion Affects Male Fertility

**FIGURE 4:**
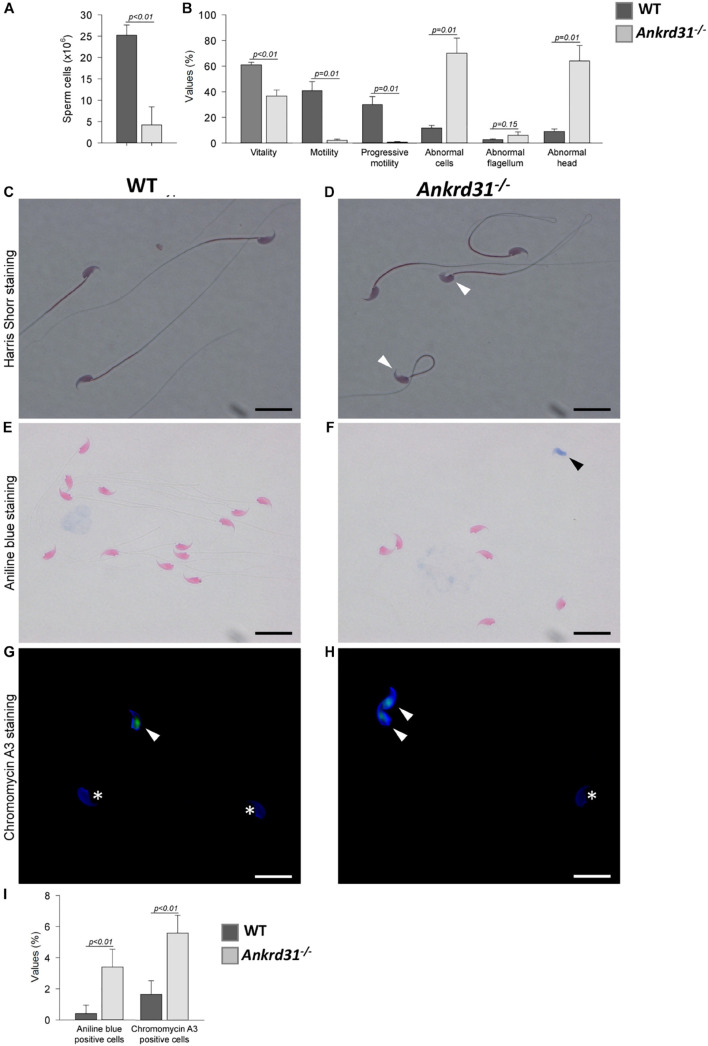
Sperm phenotyping of WT and *Ankrd31^–/–^* mice (*n* = 5 animals/each genotype). **(A)** Total sperm count (both epididymides) and **(B)** percentage values of standard parameters of WT and *Ankrd31^–/–^* mice. All percentage values were reported as mean ± SEM and compared based on student *t*-test with a probability value of less than 0.05 considered statistically significant (*p* < 0.05). **(C,D)** Harris-Shorr staining of WT and *Ankrd31^–/–^* spermatozoa (SPZ). Abnormal heads are indicated by white arrowheads. **(E,F)** Analysis of aniline blue staining of SPZ from WT and *Ankrd31^–/–^* mice. Positive staining was indicated by black arrowheads. **(G,H)** CMA3 staining of SPZ from WT and *Ankrd31^–/–^* mice. Positive staining was indicated by white arrowheads and negative staining by white asterisks. **(I)** Aniline blue and CMA3 percentage values are reported as mean ± SEM and compared based on student *t*-test with a probability value of less than 0.05 considered statistically significant (*p* < 0.05). **(C–H)** Scale bar corresponds to 10 μm.

As reported, *Ankrd31* gene deletion negatively affected germ cell progression inducing a significant reduction in SPZ production. Based on this, we decided to evaluate the possible impact on male fertility. Thus, a fertility test was carried out in WT compared to *Ankrd31^–/–^* male mice (Table 2). As shown, *Ankrd31^–/–^* male mice were totally infertile. Interestingly, when WT male mice mated with *Ankrd31^–/–^* female mice, all pregnancies were successful with a normal number of pups, suggesting that *Ankrd31* gene deletion negatively affected only male reproductive abilities. Sperm parameters were assessed from sperm cells collected from WT and *Ankrd31^–/–^ cauda* epididymides. Sperm cell number was significantly lower in *Ankrd31^–/–^* as suggested by FACS analysis (Figure 4A).

**TABLE 2:** Fertility test in *Ankrd31^–/–^* female and male mice.

Type of mating	Pregnancy	Pups	N. matings
♂WT x ♀WT	YES	7 ± 3	10
♂WT x ♀*Ankrd31^–/–^*	YES	10 ± 3	10
♂*Ankrd31^–/–^* x ♀WT	NO	–	10

*WT (n = 10) and Ankrd31^–/–^ (n = 10) male/female mice were mated to evaluate the effects of Ankrd31 gene deletion on fertility. The number of pups born from each mating was used to estimate fertility index. Only Ankrd31^–/–^ male mice were infertile.*

Sperm vitality was reduced in the *Ankrd31^–/–^* mice and correlated with a dramatic decrease of total and progressive motility (Figure 4B). The few motile SPZ exhibited severely impaired swimming [average path velocity (VAP) = 42.5 vs. 137.1 μM/s for WT mice; straight-line velocity (VSL) = 25.8 vs. 115.4 μM/s; curvilinear velocity (VCL) = 77.2 vs. 232.6 μM/s; amplitude of lateral head displacement (ALH) = 5.6 vs. 11.2 μM], concluding that *Ankrd31* gene deletion induced severe asthenozoospermia (Supplementary Videos 1,2).

In addition, a significant increase in morphological abnormalities, predominantly linked to sperm heads, was found in *Ankrd31^–/–^* SPZ (Figures 4C,D). To investigate whether the abnormal head morphology of the *Ankrd31^–/–^* sperm may be associated with nuclear abnormalities, aniline blue staining was performed to gauge chromatin quality in WT and *Ankrd31^–/–^* sperm. This staining has a selective affinity for lysine residues of histones, but not for arginine/cysteine residues of protamines. As the results showed a significant increase in the number of aniline blue-positive cells in *Ankrd31^–/–^* SPZ compared with WT SPZ (*p* < 0.05), this suggests that a percentage of the *Ankrd31^–/–^* SPZ had an immature chromatin state and an unbalanced histone/protamine ratio (Figures 4E–I).

In addition, we performed CMA3 staining on WT and *Ankrd31^–/–^ cauda* SPZ to detect potential protamine deficiency. Complementarily to the aniline blue staining, the results showed that the percentage of CMA3-positive SPZ was higher in *Ankrd31^–/–^* than in WT SPZ, suggesting that the deletion of the *Anrkd31* gene induced a reduction in protamine levels, confirming an impairment of sperm chromatin compaction (Figures 4G–I).

Finally, FISH analysis was carried out to investigate if *Ankrd31* gene deletion may induce pathological aneuploidy. Results showed that *Ankrd31^–/–^* SPZ displayed normal aneuploidy rates similar to WT mice and an equal distribution of sex chromosomes resulting in a ratio close to 1:1 (Table 3).

**TABLE 3:** Proportion of haploid, aneuploid, and diploid spermatozoa (SPZ) in wild type (WT) and *Ankrd31^–/–^* mice.

*Chromosome content*	WT	*Ankrd31^–/–^*
***Haploid***	99%	99.2%
*8,X*	49.7%	49.6%
*8,Y*	49.3%	49.6%
***Aneuploid***	1%	0.75%
*8,XY*	0.5%	0.5%
*8,XX*	0.25%	0%
*8,YY*	0.25%	0.25%
*8,8,X*	0%	0%
*8,8,Y*	0%	0%
***Diploid***	0%	0%
***Other***	0%	0%

*Three-color FISH analysis was performed on WT (n = 3) and Ankrd31^–/–^ (n = 3) sperm samples. Scoring was performed with a METAFER Metasystems^®^ device analyzing a minimum of 5,000 nuclei for each assay. Data are reported as percentage (%).*

### ANKRD31 Protein Expression in Wild-Type Epididymis

**FIGURE 5:**
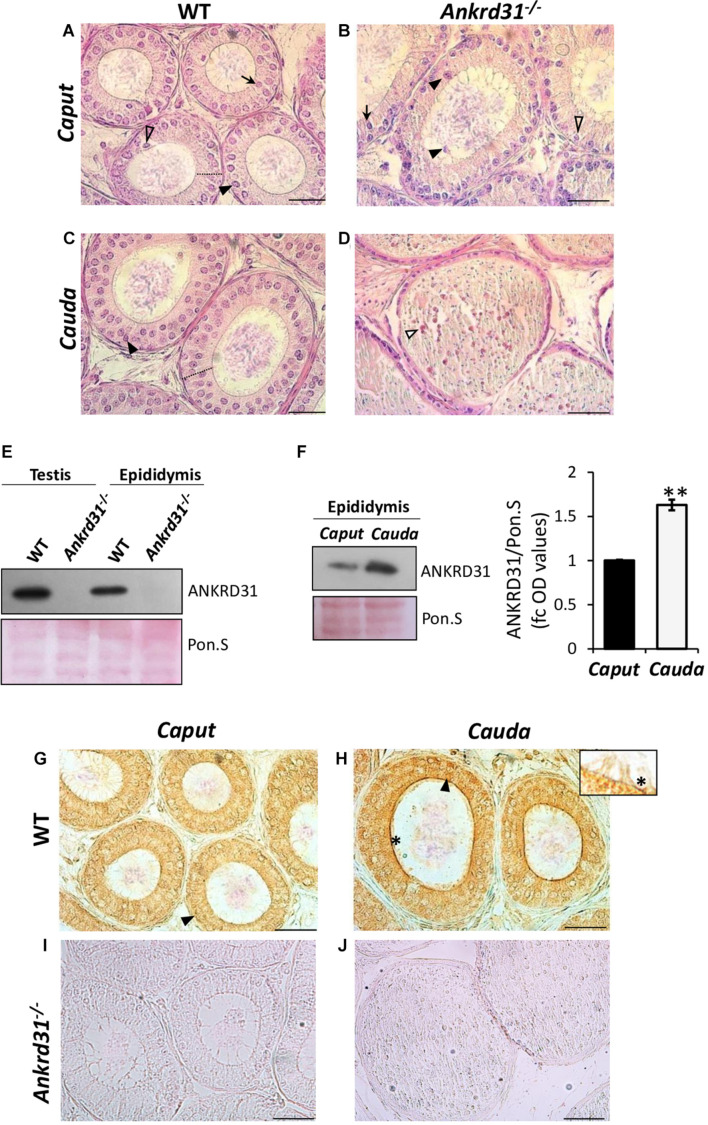
**(A–D)** H&E staining of Bouin’s fixed WT and *Ankrd31^–/–^ caput* and *cauda* epididymis sections (7 μm). Principal cells (PCs) are indicated by black arrowheads. Clear cells (CCs) are indicated by clear arrowheads. Narrow cells (NCs) are indicated by black arrows. Blood cell infiltration is indicated by white arrowheads. Columnar epithelium stratification was indicated by dashed lines. Scale bar corresponds to 50 μm. **(E)** Western blot analysis of ANKRD31 in WT and *Ankrd31^–/–^* testes and epididymides. **(F)** Western blot analysis of ANKRD31 in WT *caput* and *cauda* epididymides. Signals were quantified by densitometry analysis and normalized to Ponceau Red (Pon.S). Data are expressed in OD values as fold change and reported as mean ± SEM, **p < 0.01. **(G–J)** Immunocytochemistry of ANKRD31 in Bouin’s fixed WT and *Ankrd31^–/–^ caput* and *cauda* epididymis sections (7 μm). The ANKRD31 protein localization in PCs and stereocilia was indicated by black arrowheads and black asterisks, respectively. Scale bar corresponds to 50 μm. No ANKRD31 signal was observed in *Ankrd31^–/–^ caput* and *cauda* epididymides.

With the aim to investigate whether immotile features of *Ankrd31^–/–^* SPZ were due to epididymal alterations induced by *Ankrd31* gene deletion, histological and molecular analyses were carried out in WT and *Ankrd31^–/–^* epididymides. Histological analysis was performed by H&E staining on *caput* and *cauda* epididymal sections of both WT and *Ankrd31^–/–^* epididymides (Figures 5A–D). WT showed a pseudo-layered epithelium compared to *caput Ankrd31^–/–^* epididymides (Figures 5A,B). *Caput Ankrd31^–/–^* epididymides showed a basal detachment of principal cells in association with luminal infiltration due to a misfolding of the layered epithelium (Figure 5B). No appreciable alterations in the other epididymal epithelium cells, such as clear and narrow cells, were observed in *Ankrd31^–/–^* compared to WT in *caput* epididymides (Figures 5A,B). Instead, histological analysis of WT caudal epididymal sections showed an increased stratification of the columnar epithelium, while a severe loss of the layered structure of the epididymal epithelium was observed in *cauda Ankrd31^–/–^* epididymides where epithelial cells appeared to be not clearly distinguishable (Figures 5C,D). In addition, the caudal epididymal lumen of *Ankrd31^–/–^* was characterized by a striking infiltration of blood cells and epithelial matrix, suggesting a possible involvement of ANKRD31 protein in BEB stabilization during epididymal transit (Figure 5D).

Then, ANKRD31 protein levels were evaluated by Western blot analysis in WT and *Ankrd31^–/–^* epididymides, using the respective testis protein extracts, as internal control. As shown in Figure 5E, results confirmed the absence of ANKRD31 in both *Ankrd31^–/–^* testes and epididymides, but more interestingly, a clear ANKRD31 protein signal appeared in WT epididymides. In order to minimize any discrepancies in protein amount, Ponceau S staining was carried out. Based on these results, a regionalized analysis of ANKRD31 protein levels was carried out in WT *caput* and *cauda* epididymides by Western blot. Results showed, in *cauda* epididymides, a significant increase in ANKRD31 protein levels compared to *caput* epididymides (*p* < 0.01), suggesting an increase in ANKRD31 protein level during epididymal transit (Figure 5F). Immunocytochemistry analysis performed in WT epididymal sections using ANKRD31 antibody showed, in WT *caput* epididymides, ANKRD31 protein localization in the cytoplasm of principal cells (Figure 5G). In WT *cauda* epididymides, the ANKRD31 signal was stronger than in WT *caput* epididymides, confirming Western blot results; in addition, in *cauda* epididymides, the ANKRD31 protein localization was not only observed in the cytoplasm of principal cells, but also became more intense in stereocilia proximal to epididymal lumen (Figure 5H, inset). ANKRD31 protein immunocytochemistry was performed in *Ankrd31^–/–^* epididymal sections as internal control. As expected, no ANKRD31 signal was observed in *Ankrd31^–/–^* epididymides (Figures 5I,J).

### *Ankrd31* Gene Deletion Affects the Expression of Blood-Epididymal Barrier Junction Proteins

**FIGURE 6:**
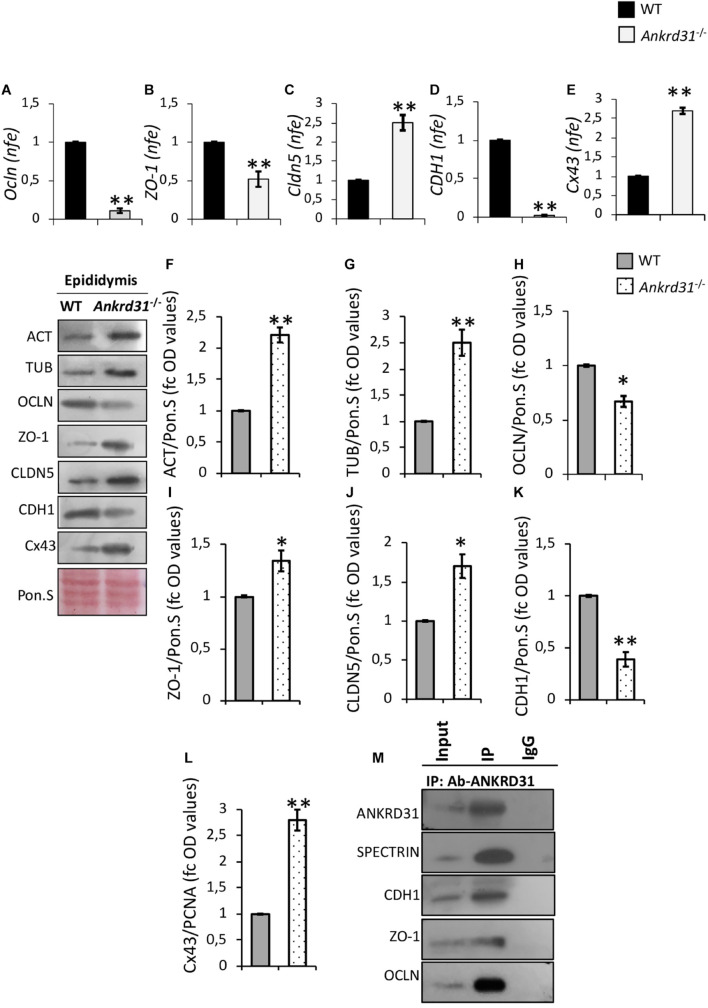
Differential expression analysis of junction epithelial mRNAs between WT and *Ankrd31^–/–^* epididymides by qRT-PCR. **(A)** *Ocln*, **(B)** *ZO-1*, **(C)** *Cldn5*, **(D)** *CDH1*, and **(E)** *Cx43* expression levels were normalized using *RP18S* as a housekeeping gene and expressed as normalized fold expression (n.f.e.). All data are reported as mean value ± S.E.M; **p < 0.01. Western blot analysis of **(F)** actin, **(G)** tubulin, **(H)** OCLN, **(I)** ZO-1, **(J)** CLDN5, **(K)** CDH1, and **(L)** Cx43 proteins levels in WT and *Ankrd31^–/–^* epididymides. Signals were quantified by densitometry analysis and normalized to Ponceau Red (Pon.S). Data are expressed in OD values as fold change and reported as mean ± SEM, *p < 0.05; **p < 0.01. **(M)** IP in WT epididymides. Total proteins collected from WT epididymides were immunoprecipitated using ANKRD31 antibody. Protein interaction among ANKRD31, SPECTRIN, CDH1, ZO-1, and OCLN was detected by Western blot analysis.

In order to study the effects of *Ankrd31* gene deletion on BEB integrity, the expression analysis of epithelial junction markers, in WT and *Ankrd31^–/–^* epididymides, was carried out by qRT-PCR (Figure 6). Regarding tight junctions, results showed lower expression levels of *Ocln* and *ZO-1* in *Ankrd31^–/–^* than WT epididymides (Figures 6A,B) (*p* < 0.01) and, conversely, higher expression levels of *Cldn5* in *Ankrd31^–/–^* than WT epididymides (*p* < 0.01) (Figure 6C). For adherens and gap junctions, a reduction in *CDH1* and an increase in *Cx43* expression levels (*p* < 0.01) were observed in *Ankrd31^–/–^* compared to WT epididymides (Figures 6D,E), suggesting a complete deregulation of BEB epithelial junctions.

Western blot analysis was carried out to investigate the levels of junction epithelial proteins between WT and *Ankrd31^–/–^* epididymides. Interestingly, actin and tubulin protein levels, frequently chosen as housekeeping proteins to normalize protein signals, were higher in *Ankrd31^–/–^* than WT epididymides (*p* < 0.01), suggesting possible cytoarchitecture anomalies of epididymal epithelial cells (Figures 6F,G). For this reason, protein profiles were normalized relative to Ponceau S. In *Ankrd31^–/–^*, a significant reduction in OCLN and an increase in ZO-1 and CLDN5 protein levels were observed in comparison to WT epididymides (Figures 6H–J; *p* < 0.05). In addition, quantitative densitometry analysis of CDH1 and Cx43 signals, showed, respectively, lower and higher protein levels in *Ankrd31^–/–^* than WT epididymides (Figures 6K,L; *p* < 0.01), suggesting that *Ankrd31* gene deletion negatively affected BEB junction epithelial protein levels.

Based on these results, protein IP experiments, using ANKRD31 antibody, were carried out in WT epididymides to investigate a possible physical interaction between ANKRD31 and junction epithelial proteins. As reported, ANKRD31-IP showed protein signals for CDH1, ZO-1, and OCLN, suggesting that the deregulation of BEB observed in *Ankrd31^–/–^* epididymides may be the direct consequence of the loss of physical interaction among ANKRD31 and junction epithelial proteins (Figure 6M). In addition, ANKRD31-SPECTRIN interaction was evaluated as a positive experimental control in order to demonstrate the efficiency in the ANKRD31 IP assay (Figure 6M).

## Discussion

ANKRD are a family of proteins characterized by 24 tandem ankyrin repeats useful for the recognition and the attachment of integral membrane proteins to the cytoskeleton. Structurally, four functional domains have been identified: (i) the N-terminal domain containing tandem repeats; (ii) the central spectrin-binding domain; (iii) a second central domain involved in apoptosis, and (iv) the regulatory C-terminal domain (Bennett and Baines, 2001; Michaely et al., 2002). The binding between ankyrin and spectrin, and, in turn, between spectrin and actin, is required to ensure the integrity of the plasma membrane. In this regard, the intricate mechanobiological pathway, involving plasma membrane-cytoskeletal and nuclear proteins, has a fundamental role in the propagation of mechanical signals from the membrane to the nucleus with the regulation of chromosome positioning and gene expression, as a consequence (Rowat et al., 2006; Lombardi et al., 2011). Recent findings highlight growing evidence about the involvement of these mechanical forces during spermatogenesis to ensure a proper sperm architecture (Kmonickova et al., 2020; Manfrevola et al., 2021). Mechanical forces participate during: (i) the meiotic recombination to support chromosome pairing and telomere attachment; (ii) the spermiogenesis phase to allow a correct head/tail sperm formation and interconnection (Ding et al., 2007; Göb et al., 2010; Pasch et al., 2015; Manfrevola et al., 2021). In this context, recent literature reported new findings regarding the involvement of ANKRD proteins in spermatogenesis. In particular, ANKRD31 is involved in the correct meiotic double-strand breaks formation, acting as a protein scaffold needed to anchor several chromosome recombination factors. The generation of *Ankrd31^–/–^* mice by a CRISPR-Cas9 strategy against exon 3 of the *Ankrd31* gene led to complete male sterility depending on meiotic recombination defects that induced spermatocytes apoptosis and meiotic arrest (Boekhout et al., 2019; Acquaviva et al., 2020). Papanikos et al. (2019) generated *Ankrd31^–/–^* male mice against exon 6 of the *Ankrd31* gene. In agreement with Boekhout et al., they confirmed the central role of ANKRD31 in the double-strand break machinery complex as a key factor to ensure recombination in the pseudoautosomal regions which permits correct sex chromosome segregation (Papanikos et al., 2019). Both Papanikos and Boekhout, generating mutations in exon 6 and exon 3 of the *Ankrd31* gene, respectively, observed the impact of these mutations on female fertility; in detail, exon 6 mutation generated fertile females that lost fertility faster than WT females in advanced age (Papanikos et al., 2019). Instead, exon 3 mutation generated females with greatly reduced oocyte numbers at all the ages examined (Boekhout et al., 2019). Here, exon 4 mutation did not induce any effect on female fertility, suggesting that such a phenotypic difference may depend on the specific exon deleted.

Interestingly, the *Ankrd31^–/–^* model generated by exon 6 mutation showed elevated apoptosis in early spermatocytes similar to *Ankrd31^–/–^* male mice generated by exon 3 mutation, but, differently, a fraction of spermatocytes was able to progress to the first meiotic metaphase, where they arrested.

Here, we generated an *Ankrd31^–/–^* male model, with the same strategy, against exon 4 of the *Ankrd31* gene and characterized its reproductive phenotype.

Macroscopic analysis showed a significant reduction in *Ankrd31^–/–^* testis weight suggesting an impaired spermatogenesis. Morphological analysis carried out on testis sections showed a deregulation of normal seminiferous epithelium organization. Interestingly, our *Ankrd31^–/–^* model showed typical meiotic arrest observed in the other *Ankrd31^–/–^* models, but sporadic spermatids were observed. FACS analysis of germ cell populations isolated from *Ankrd31^–/–^* testes confirmed the increase in spermatogonia and spermatocyte populations and showed a reduction in spermatid population, but not its total absence. As Papanikos et al. (2019) showed in their *Ankrd31^–/–^* model that few spermatocytes progressed to the first meiotic metaphase, our data lead us to hypothesize that, despite the impaired meiotic phase, some post-meiotic germ cells were able to progress and differentiate in SPZ.

Based on this, we decided to isolate SPZ from *cauda* epididymides and analyze sperm qualitative parameters using a computational sperm analyzer. *Ankrd31^–/–^* SPZ showed decreased total and progressive motility associated with sperm head morphological abnormalities. The complete absence of motile SPZ explained why *Ankrd31^–/–^* male mice were totally infertile, unlike *Ankrd31^–/–^* females. The analysis of histone and protamine content, using aniline blue and CMA3 staining, respectively, indicated that abnormal sperm head morphology of *Ankrd31^–/–^* SPZ was partially associated with an incorrect protamination status, probably due to a defective histone displacement occurring in the spermiogenesis phase (Chioccarelli et al., 2020).

Overall, unlike Boekhout et al. (2019) we observed that few SPZ, abnormal for morphology and motility, were produced and released in the epididymis. In order to understand if the affected sperm motility in *Ankrd31^–/–^* mice may be a consequence of epididymal anomalies too, we evaluated the epididymal epithelium given its importance in the maintenance of the luminal microenvironment useful for sperm motility acquisition (Cornwall, 2009; Sullivan, 2015; Breton et al., 2016; Zhou et al., 2018). Histological analysis showed that, physiologically, the columnar epididymal epithelium undergoes a pluri-stratification development, from *caput* to *cauda*, depending on a well-structured organization of principal cells. The *Ankrd31^–/–^* epididymal epithelia lacked this caudal cellular organization, thus suggesting a possible correlation between these anomalies and the absence of ANKRD31 protein. Here we evaluated, for the first time, the expression of ANKRD31 in WT epididymides that, increasing during epididymal transit, was strongly localized in principal cells. Based on this, we hypothesized that, physiologically, epididymal ANKRD31 protein participates in the correct functioning of the BEB necessary to create the optimal microenvironment involved in epididymal sperm maturation and motility. A mature epididymal BEB formation, in turn, strongly depends on epithelial intercellular junction complexes, including tight, adherens, and gap junctions (Cyr et al., 1992, 2007; Cyr, 2011).

The molecular analysis of the principal proteins composing epididymal intercellular junction complexes in *Ankrd31^–/–^* epididymides showed a deregulated expression of these markers, in both mRNAs and proteins, that well matched with the disorganized epididymal epithelium observed by histological analysis. However, mRNA and protein abundances were not perfectly correlated with each other. The correlation between these two classes of biological molecules is deeply influenced by several biological factors as well as by methodological constraints, and frequent experimental discrepancies are typically attributed to other levels of regulation between transcript and protein products (Maier et al., 2009; Vogel and Marcotte, 2012).

In addition, we showed a protein interaction among ANKRD31 and junction epithelial proteins such as CDH1, ZO-1, and OCLN. Our results are consistent with previous findings demonstrating a physical interaction between ANKRD proteins and several junction epithelial proteins in order to stabilize and place them in specific cellular areas (Kizhatil et al., 2007; Cadwell et al., 2016). Taken together, these observations lead us to speculate that ANKRD31 interacts with junction epithelial proteins and that BEB maturity and stability appeared to be compromised as a direct consequence of the loss of ANKRD31 within specific epithelial epididymal cell types.

Some aspects about the role of ANKRD31 during spermatogenesis still need to be clarified. In the this work, four fundamental points should be highlighted: (i) we confirmed that *Ankrd31* gene deletion negatively affected spermatogenesis at the meiotic level, as recently reported; (ii) in our *Ankrd31^–/–^* model, despite the impaired germ cell progression, few abnormal SPZ were produced and released into the epididymal lumen, unlike other *Ankrd31* mutant models; (iii) the released SPZ were motile in a very low percentage due to a compromised BEB depending on the lack of interaction among ANKRD31 and the intercellular junction proteins; and (iv) sperm defects observed may be a secondary consequence of both early meiotic arrest and BEB dysfunction.

## Data Availability Statement

The raw data supporting the conclusions of this article will be made available by the authors, without undue reservation.

## Ethics Statement

Animal care and experimental procedures were performed following French and European guidelines on the protection of animals used for scientific purposes and approved by an ethical committee for animal experimentation (CEEA Val de Loire Projet 12884).

## Author Contributions

RC, FM, and FG: conceptualization and formal analysis and investigation. FM, GM, CC, DR, KR, YL, PF, LB, DA, and FG: methodology and figure preparation. FM, GM, PF, YL, and FG: writing–original draft preparation. RC, RP, and FG: writing–review, editing, and funding acquisition. RC and FG: supervision. All authors contributed to the article and approved the submitted version.

## Conflict of Interest

The authors declare that the research was conducted in the absence of any commercial or financial relationships that could be construed as a potential conflict of interest.

## Publisher’s Note

All claims expressed in this article are solely those of the authors and do not necessarily represent those of their affiliated organizations, or those of the publisher, the editors and the reviewers. Any product that may be evaluated in this article, or claim that may be made by its manufacturer, is not guaranteed or endorsed by the publisher.

## Footnotes

1
http://www.ncbi.nlm.nih.gov/tools/primer-blast/

## Supplementary Material

Supplementary Video 1 Analysis of sperm motility in WT (n = 3) and *Ankrd31^–/–^* (n = 3) mice.

Supplementary Video 2 A minimum of 100 motile SPZ was recorded in real time and analyzed for each sample.
